# Malaria in children and women of childbearing age: infection prevalence, knowledge and use of malaria prevention tools in the province of Nyanga, Gabon

**DOI:** 10.1186/s12936-020-03411-5

**Published:** 2020-11-02

**Authors:** Roméo Karl Imboumy-Limoukou, Sydney Maghendji-Nzondo, Pater Noster Sir-Ondo-Enguier, Julie Niemczura De Carvalho, Nathalie Pernelle Tsafack-Tegomo, Julie Buekens, Alain Prince Okouga, Augustin Mouinga-Ondeme, Sylvie Kwedy Nolna, Jean-Bernard Lekana-Douki

**Affiliations:** 1 Unité Evolution, Epidémiologie et Résistance Parasitaire (UNEEREP), Centre Interdisciplinaire de Recherches Médicales de Franceville (CIRMF), Franceville, BP: 769 Gabon; 2 Departement d’Epidémiologie Biostatistiques et Informatique Médicale, Université des Sciences de la Santé, Libreville, BP: 4009 Gabon; 3 Unité des Infections Rétrovirales et Pathologies Associées, Centre Interdisciplinaire de Recherches Médicales de Franceville (CIRMF), Franceville, BP: 769 Gabon; 4 Capacity of Leadership Excellence and Research (CLEAR), Yaounde, Cameroon; 5 Medical Care Development International (MCDI), Silver Spring, Maryland, 20910 USA

**Keywords:** Malaria, Prevalence, Preventive measures, Pregnant women, Children, Gabon, Nyanga

## Abstract

**Background:**

There is little information on the social perception of malaria and the use of preventative measures in Gabon, especially in rural areas. Adequate knowledge of malaria prevention and control can help in reducing the burden of malaria among vulnerable groups, particularly pregnant women and children under 5 years old living in malaria-endemic settings. This study was designed to assess the prevalence of malaria and the knowledge and attitude towards this disease in households in Nyanga Province.

**Methods:**

A cross-sectional study was conducted to assess malaria knowledge, prevention practices and prevalence of the malaria infection in five departments of Nyanga Province. Plasmodial infection was diagnosed in children  ≤ 5 years of age and women aged 15-49 years using rapid diagnostic tests. A questionnaire was administered randomly to women aged 15–49 years and to the parents or guardians of children aged ≤ 5 years in 535 households during a 2-week period in March 2018. Overall, the respondents’ socio-demographic characteristics, knowledge of malaria, malaria prevention practices and malaria prevalence were evaluated and compared across the five departments.

**Results:**

Data from a total of 1,307 participants were included in this study, including 631 women of childbearing age (61 of them pregnant) and 676 children. Practically the entire (97.7%) interviewed population had heard about malaria and attributed the cause of malaria to a mosquito bite (95.7%). This survey revealed that the reported rate of reported bed-net use was 73.3%. The study observed an average malaria parasite prevalence of 13.9%. All departmental capitals of Nyanga Province had a significant level of malaria infection except for Mayumba where no plasmodial infection was found.

**Conclusion:**

High malaria prevalence is found in the departmental capital cities of Nyanga Province. This study reveals that respondents have a high knowledge of the malaria symptoms, its mode of transmission and preventive measures. Despite this high level of knowledge of the disease and its preventive measures, the incidence of malaria remains relatively high in this rural community highlighting the need for other types of interventions.

## Background

In 2019, malaria remained the deadliest parasitic disease for human beings. An estimated 228 million cases of malaria occurred worldwide in 2018, most of which were in the WHO African Region (200 million or 92%). The same year, 405,000 malaria-related deaths were recorded. Children under 5 years old are the most vulnerable group affected by malaria; in 2017 they accounted for 61% (266,000) of all malaria deaths worldwide [1]. It is now known that pregnant women are more susceptible to malaria than their non‐pregnant peers [2]. Malaria during pregnancy is responsible for serious consequences for both the mother and her child, among which fetal growth restriction, prematurity and still birth contribute to perinatal and neonatal mortality  [3]. In areas where the intensity of transmission is moderate to high, leading to higher levels of acquired immunity, most falciparum malaria infections during pregnancy remain asymptomatic and are frequently undiagnosed and untreated [4]. The 2018 World Malaria Report also reveals insufficient levels of access and adhesion to lifesaving malaria prevention tools and interventions. A considerable proportion of people at risk of infection are not being protected, including pregnant women and children in Africa [1] .

Gabon is a hyperendemic area in which malaria burden fluctuates. Transmission is perennial since the equatorial climate favours mosquito proliferation and larval development. However, over the last decade, Gabon has gradually strengthened malaria control interventions. Changes in the national anti-malarial policy, such as the introduction of artemisinin-based combination therapy (ACT) as first-line treatment in all public facilities, the introduction of malaria rapid diagnostic tests (RDTs), the distribution of impregnated bed nets, and the implementation of intermittent preventive treatment during pregnancy have led to a decline in the malaria burden in urban areas [5]. After the implementation of ACT in 2005, a decrease in malaria burden was observed, but for the past few years, a recrudescence was noted in the urban areas of Franceville and Libreville. However, prevalence did not change in rural areas [6].

Nyanga is the southernmost province among Gabon’s 9 provinces with a population of 52,854 inhabitants. The provincial capital is Tchibanga, which had a total of 31,789 inhabitants in 2013 (more than half of the total population in the province). Nyanga is the least populated province of the 9 provinces and the second least developed, following Ogooué-Ivindo. Nyanga Province has poor economic development, few industries are present and it is surrounded by forest and crossed by several rivers. Nyanga is divided into 6 departments: Basse-Banio (7,192 inhabitants), Douigny (5,235 inhabitants), Doutsila (4,331 inhabitants), Haute-Banio (1,413 inhabitants), Mongo (2,602 inhabitants), and Mougoutsi (31,789 inhabitants).

As malaria control interventions are increased, rational approaches are needed to monitor their impact over time. However, the prevalence of malaria has not been well described in rural Nyanga Province as very little data on the epidemiology of malaria are available. Furthermore, little effort has been placed on examining inhabitants’ knowledge regarding malaria transmission modes and the preventive methods used and practiced among people living in rural communities, particularly in Nyanga Province.

To truly fight malaria in the world, a comprehensive approach that includes vector control measures and early diagnosis and treatment, especially at village level, is needed. Using this approach, the American NGO Medical Care Development International (MCDI) has been able to significantly reduce the burden of malaria in Equatorial Guinea in recent years [7]. This study piloted an active screening approach to monitor malaria prevalence in two target populations: women of childbearing age (including pregnant women) and children aged 5 years or under. Additionally, their knowledge of malaria was evaluated as well the behaviours of children’s parents or guardians and of the women regarding the disease in Nyanga Province.

## Methods

### Population and study sites

**Fig. 1:**
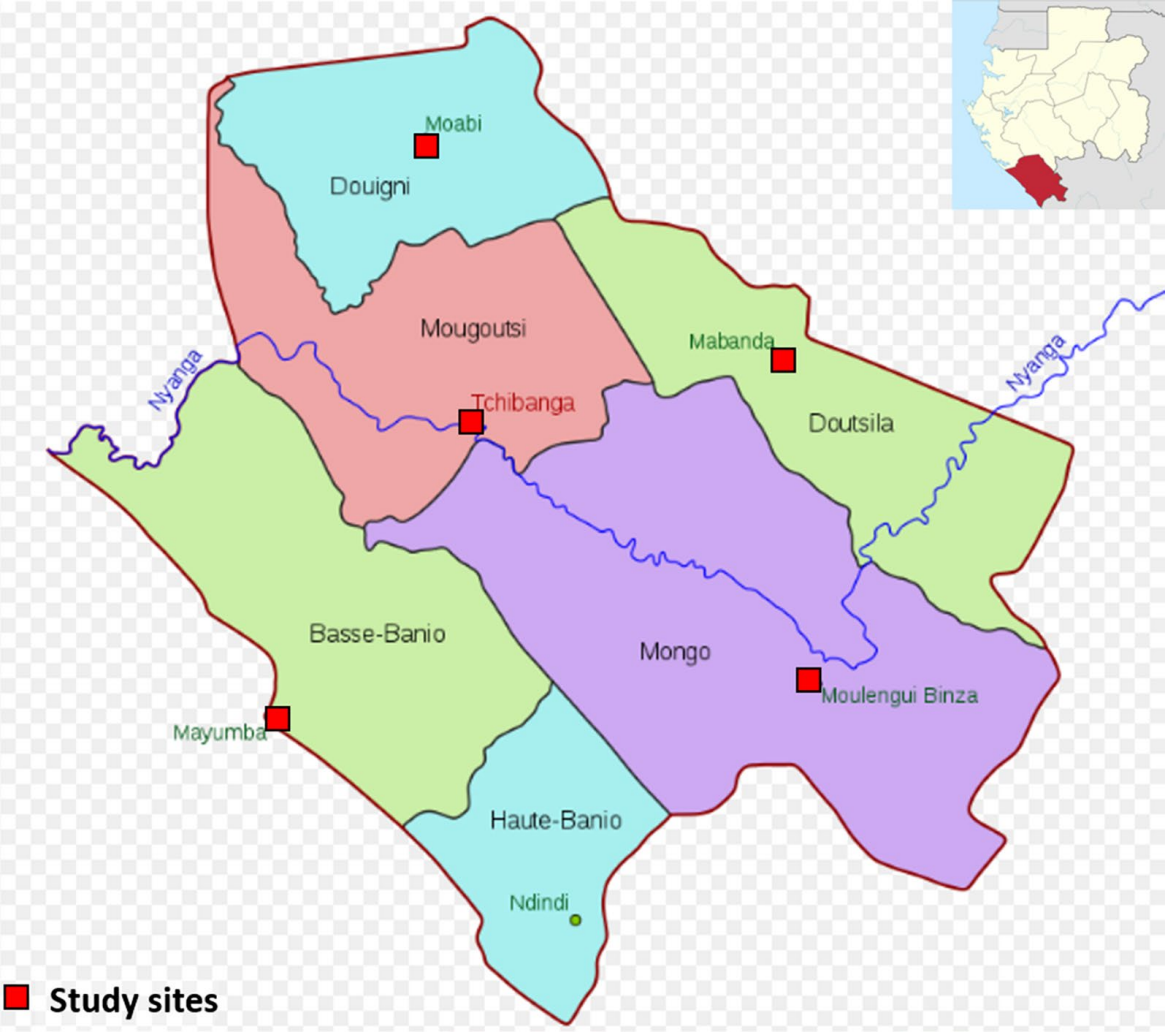
Study sites

This community-based, cross-sectional, descriptive and analytical epidemiological study was conducted from 18 to 27 March, 2018 in the 5 department capitals of Nyanga Province, located in southern Gabon, to assess malaria prevalence as well as the population’s basic knowledge of malaria and their behaviour regarding prevention. The cities included in this study were: Mayumba, Moabi, Mabanda, Moulengui-Binza, and Tchibanga, in the departments of Basse-Banio, Douigny, Doutsila, Mongo and Mougoutsi, respectively (Fig. 1).

The studied population was composed of children aged 5 years or under and women of childbearing age. Inclusion criteria for sampling eligible participants stipulated that participants must: (1) reside within the study site during the study period; (2) have signed the consent form; 3) be children aged 5 years old or under, and be women aged 15–49 years. All women aged 15–49 years old and children aged 5 years or under in the households surveyed were included in the study. The study was approved by the Gabonese National Ethics Committee (N° 001/PR/SG/CNE/2018).

### Sampling of study participants and collection of data on malaria knowledge and use of preventive measures against malaria and level of knowledge of participants

Before sampling, households with children ≤ 5 years of age and women of childbearing age were identified. A random sample of households was selected for inclusion in the study; all neighbourhoods of the departmental capitals were represented. If no member from a selected household was willing to participate, the teams approached the next house on the list until reaching a willing household. The households visited were marked with chalk. The survey was performed by 10 teams of 3 persons deployed across the 5 cities. The sample size was not calculated as an open sampling method was used.

Data on the use of bed nets and insecticides, and some information on education and knowledge of malaria were collected with a questionnaire. An additional file shows the questionnaire in more detail (Additional file 1). The parents/guardians of children under 5 years old, woman of childbearing age and pregnant women were asked whether they slept under bed nets and whether they sprayed insecticide in their houses. To determine their level of education on malaria, some key questions were asked, such as: “Have you ever heard of malaria?”, “How is malaria transmitted?”, “Do you know the modes of transmission of malaria?”, “How could you avoid malaria?’’, and “What are the symptoms of malaria”. Participants that responded correctly to these questions were considered as having a basic knowledge of malaria.

### Malaria parasite detection and treatment

After obtaining the consent of all recruited participants, a RDT was performed. Capillary blood was obtained via finger stick for malaria testing using RDT SD BIOLINE Malaria Ag P.f/Pan test (Abbott, USA). In different visited households, malaria infection was diagnosed only in children ≤ 5 years of age and women aged 15–49 years. Test results were recorded on a patient sheet and data on symptoms were also collected. Participants with positive RDT results were offered immediate treatment with either artemether-lumefantrine for children and pregnant women in their second or third trimester Coartem^®^, child: 20 mg/120 mg and adult: 80 mg/480 mg (Novartis, Switzerland), or quinine for pregnant women in their first trimester Surquina 250 mg (Innothera Chouzy, France), according to national treatment guidelines.

### Data analysis

Statistical analyses were carried out with Epi-info version 3.5.3 (2005, CDC, Atlanta, USA) and STATA version 14 (Stata Corp, College Station, USA). Age was expressed as the mean and standard deviation. The age variable was transformed into a categorical variable with 6 classes: 15–20 years, 21–25 years, 26–30 years, 31–35 years, 36–40 years, and 41–49 years. The Chi square test was used to compare categorical variables. Values of *p* < 0.05 were considered to indicate a statistically significant difference.

## Results

A total of 1,307 participants were recruited in this study, originating from 535 homes. Included in the study were 631 women of childbearing age, including 61 pregnant women, and 676 children. Data were available only for 1,296 participants. The pregnancy status and the number of months of pregnancy of the women were self-reported and no confirmatory pregnancy tests were carried out.

### Clinical and socio-demographic characteristics of the studied population

All participants were residents of Nyanga Province. The mean age of interviewed study participants was 27.8 ± 11 months. On average, there were 7.45 ± 3.50 people per household, including 1.95 ± 1.34 children and 0.17 ± 0.43 pregnant women with an average of 5.51 ± 2. 58 months of pregnancy. About 162 out of 1,299 (12.5%) were febrile (axillary temperature > 37.5 °C) and 7.5% (97/1296) had history of fever in the 14 days prior to sampling. The general description of participants is shown in Table 1.

**Table 1:** Socio-demographic characteristics of study population

Variables	Cities (%; n/N)					Total
	Mabanda	Mayumba	Moabi	Moulingui-Binza	Tchibanga	
Categories						
Children (≤ 5 years)	46.2% (42/91)	60.6% (114/188)	53.7% (87/162)	57.5% (50/87)	49.2% (383/779)	676
Women of childbearing age (15–49 years)	53.9% (49/91)	33.5% (63/188)	40.1% (65/162)	39.1% (34/87)	46.1% (359/779)	570
Pregnant women (15–49 years)	0.0% (0/91)	5.9% (11/188)	6.2% (10/162)	3.5% (3/87)	0.4% (37/779)	61
Mean age ± SD (years)	16,8 ± 15,2	15,6 ± 14,7	15,7 ± 13,7	15,1 ± 15,8	16,0 ± 14,7	
Women occupation (N = 620)						
Student	9.5% (4/42)	1.4% (1/72)	36.0% (27/75)	16.2% (6/37)	20.8% (82/394)	120
Worker	38.1% (16/42)	30.5% (22/72)	33.3% (25/75)	48.7% (18/37)	23.4% (92/394)	173
Unemployed	52.4% (22/42)	68.1% (49/72)	30.7% (23/75)	35.1% (13/37)	55.8% (220/394)	327

### General knowledge on malaria causes, symptoms and prevention

Almost the entire interviewed population previously heard about malaria (97.7%) and attributed the cause of malaria to a mosquito bite (95.7%, 617/649). The majority of the respondents (96.2%, 624/649) also thought that they could get malaria by walking in the rain. Some respondents (17.3%, 112/649) believed malaria to be transmitted by ingesting of dirty water, and finally, (8.3% 54/649) of the studied population thought that malaria was transmitted sexually. The most commonly known clinical symptoms by the respondents were fever (88.7%), body aches (78.3%), headache (74.1%), fatigue (71.3%), vomiting (49.69%), cough (21.9%), diarrhoea (20.1%), and stomach aches (19.8%).

Knowledge and action for malaria prevention and vector control were also evaluated. The majority of the interviewed individuals thought that the use of bed nets at home and environmental sanitation (95.06 and 84.57%, respectively) could prevent malaria. More than half of the respondents thought that spraying insecticides could also prevent malaria (64.7%). Fewer than half of the respondents thought that vaccination and washing their hands before eating (64.5 and 49.9%, respectively) could also be ways to prevent malaria.

### Information source

The interviewed people who had already heard of malaria reported receiving information on malaria from one or more sources. The sources of information were varied and are reported in Table 2. Among the different sources of information, the largest proportion of respondents had received information regarding malaria from a media source (67.7%), followed by hospitals, health centre or doctors (22.9%). The sources least reported were schools and sensitization campaigns (2.6%). Among media sources television and social media were a major source of malaria information for all respondents.

**Table 2:** Sources of information regarding malaria among respondents

Source of information	Frequency, N = 649
Media sources^a^	64.7% (420)
Hospital, healthcare center	22.9% (149)
Discussion	6.9% (45)
School	2.6% (17)
Awarness campaign	2.6% (17)

^a^Television, radio, newspaper, books, social media, internet, poster

### Preventive measures

This survey revealed that the rate of reported bed-net use was 73.3% (924/1,260). Insecticide spraying was used by 52.1% (574/1,103) of the subjects. Fans were more commonly used according to this study than air-conditioners (58.4 *vs* 3.2%, p < 0.05). More than half of the women had not received an insecticide-treated bed net (ITN) during antenatal visits in their last pregnancy. Sixty-six per cent (66.1%) of women said that long-lasting insecticide nets (LLINs) are free in public health facilities, but 26.1% reported that they are not free. As for intermittent preventive treatment with sulfadoxine-pyrimethamine (IPT-SP), 50.2% of women said that IPT-SP is free in public health facilities, whereas 32.6% of them stated it was not.

### Characteristics of plasmodial infection

**Fig. 2:**
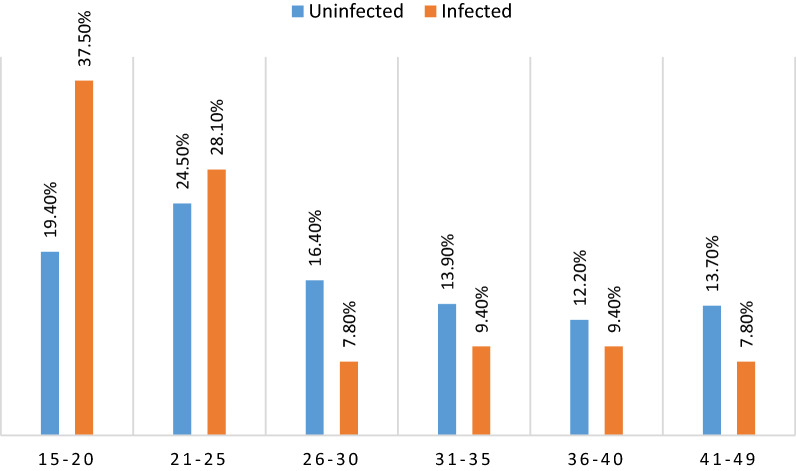
Comparison between infected and uninfected women of childbearing age

**Fig. 3:**
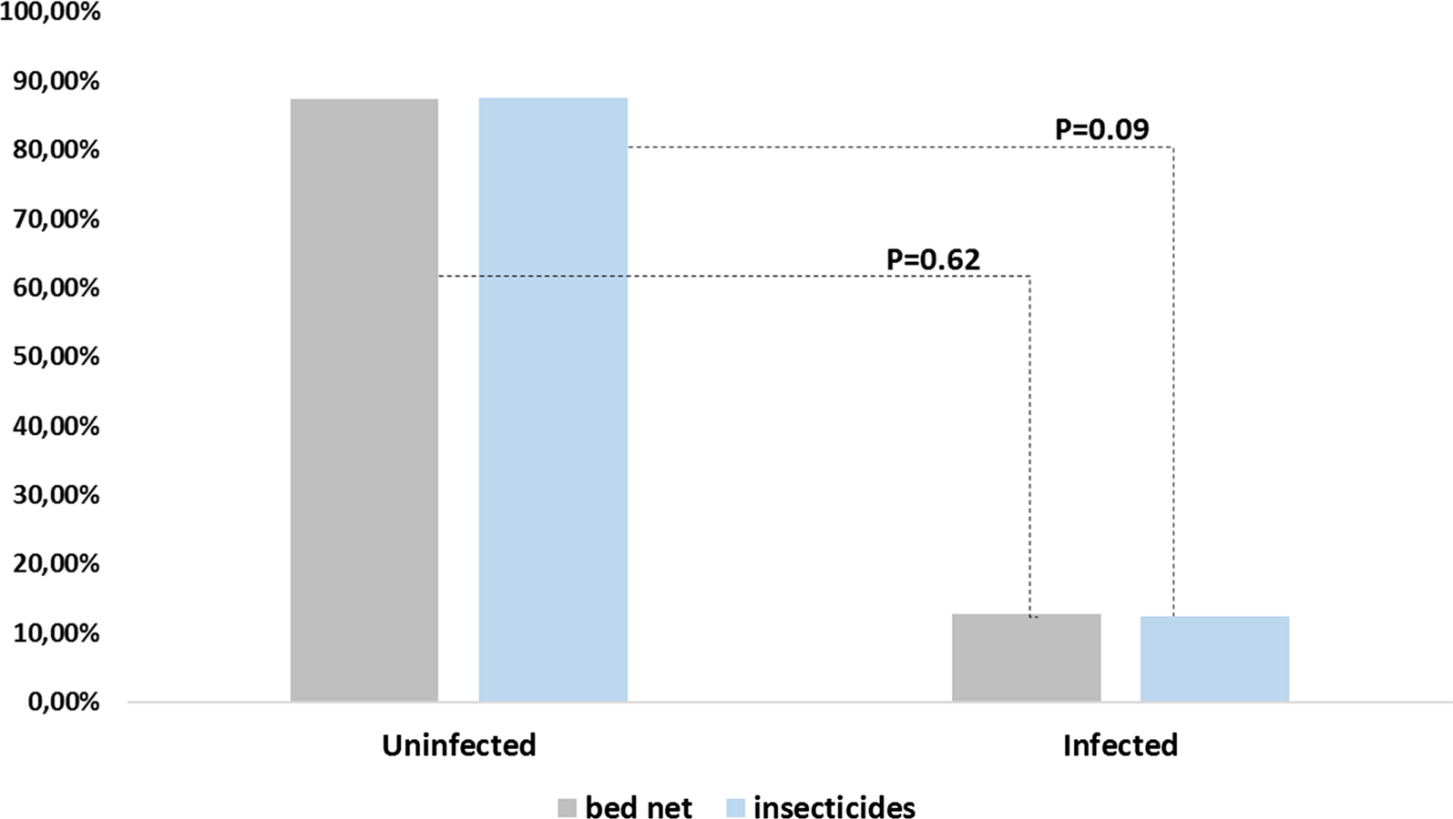
Comparison of preventive measures between infected and uninfected individuals

The overall prevalence of plasmodial infection diagnosed with RDT was 13.9% (180/1,296). It was significantly different between department capitals (p < 0.05). The prevalence of *Plasmodium* infection was higher in Tchibanga (8.7%, 145/776) compared to Moulingui-Binza (9.2%, 8/87), (p = 0.02) and Mabanda (3,6%, 3/83), (p = 0.0005). In Moabi, the prevalence of plasmodial infection (14.8%, 24/162) was higher than in Mabanda (3.6%, (3/83), (p = 0.008). There was no plasmodial infection diagnosis in Mayumba. The overall prevalence of plasmodial infection was 16.5% (111/674) in children ≤ 5 years old, 10.2% (57/558) in women of childbearing age, and 20.0% (12/60) in pregnant women. The difference in prevalence of plasmodial infection between children, women of childbearing age, and pregnant women was significant in the surveyed sites (p = 0.01). The prevalence of plasmodial infection between these same groups was also significantly different in Tchibanga and Moabi (p = 0.007, p = 0.006, respectively). In addition, the prevalence of infection was significantly different between age groups in women of childbearing age. Women between 11 and 20 years of age (37.50%) were more infected than women in other age groups (*p *= *0.012*). Table 3 and Fig. 2 show a summary of the general characterization of the plasmodial infection by department capitals. There was no statistically significant difference in the rates of bed-net use and of insecticide use between infected and uninfected study participants (p > 0.05), as shown in Fig. 3.

**Table 3:** Comparison between infected and uninfected women of childbearing age

	Cities (%; n/N)					
	Mabanda	Mayumba	Moabi	Moulingui-Binza	Tchibanga	Total
Parameters						
Positive RDT (%; n/N)	3.6% (3/83)	0.0% (0/188)	14.8% (24/162)	9.2% (8/87)	18.7% (145/776)	180
Mean age ± SD (years)	*16.8 *± 15.2	*1.6 *± 14.7	15.7 ± 13.1	15.1 ± 15.8	16.0 ± 14.7	
Categories						
Children (≤ 5 years)	4.9% (2/41)	0.0% (0/114)	23.0% (20/87)	12.0% (6/50)	21.7% (83/382)	111
Women of childbearing age (15–49 years)	2.4% (1/42)	0.0% (0/63)	6.2% (4/65)	2.9% (1/34)	14.4% (51/354)	57
Pregnant women (15–49 years)	–	0.0% (0/11)	0.0% (0/10)	33.3% (1/3)	30.6% (11/36)	12
Women occupation (N = 68)						
Student	–	–	50.0% (2/4)	50.0% (1/2)	19.7% (12/61)	15
Worker	–	–	25.0% (1/4)	–	19.7% (12/61)	13
Unemployed	100% (1/1)	–	25.0% (1/4)	50.0% (1/2)	60.7% (37/61)	40

## Discussion

This study provides the first data on the epidemiological parameters, characteristics of plasmodial infection, and the knowledge and prevention practices regarding malaria in a vulnerable population of Nyanga Province in Gabon. In this cross-sectional survey, 1,307 individuals composed of women of childbearing age and children aged 5 years or under were included. The vast majority of respondents (97.7%) in Nyanga Province (rural area) were aware of malaria. Similar results have been reported in other studies in other countries [8–11].

In the present study, data revealed that study population had adequate knowledge regarding the mosquito bite as the means of transmission. However, a large proportion of respondents also falsely thought that walking in the rain was another cause of malaria. The questionnaire may have failed to capture causes other than the four indicated. Results of this work also demonstrate that fever and headache, fatigue and aches were correctly identified as symptoms of malaria, which corroborates results from similar studies conducted in Cameroon, Ethiopia and Tanzania [8, 9, 12] where respondents were able to name at least one symptom of malaria.

National malaria programmes need to know how and where their populations obtain information regarding malaria in order to better plan their communication activities. This study revealed that most of the population obtain information on malaria from media sources and, to a lesser extent, from hospitals and health centres. Given the young mean age of the interviewed study participants (28 ± 10.8 years), these results are to be expected as, in Gabon, the majority of young people watch television and are connected to social media via the internet. These findings are different from those obtained in another study led in India [13] in which overall, hospitals, health centres or doctors were the most frequently cited interpersonal source of information about malaria. This disparity could be explained by the difference in age of the women included in the different studies. The low prevalence of sensitization campaigns could be misleading as such campaigns used a variety of communication channels (i.e., medias sources, communication in school), among others. It is possible that the respondents heard about malaria through these sources without necessarily associating them with the campaigns. These results also highlight the need for public health professionals to work together with media, as well as hospital facilities to disseminate accurate and reliable malaria information and use age-appropriate means of communication in order to have the most impact.

Respondents showed adequate knowledge with regard to bed nets, spraying of insecticides and environmental sanitation as effective prevention strategies. Over 95% of people interviewed in Nyanga Province reported using bed nets to prevent malaria. Indeed, in Gabon and in other countries of Central Africa, the national policy against malaria relies on the use of ITNs. During this last decade, a concerted campaign against malaria has led to unprecedented levels of interventions across sub-Saharan Africa [14]. African governments and decision-makers in the health sector are conducting mass awareness campaigns through audiovisual networks, social networks and newspapers. School-age children are usually educated about malaria in school. Although these campaigns take place throughout the year, they are emphasized during internationally celebrated Malaria Awareness Day. Also, in Gabon, as other malaria-endemic countries, ITNs are freely distributed to pregnant women and children under 5 years, as is intermittent preventive treatment for pregnant women; indoor residual spraying is also widely used across Africa with increasing amounts of coverage achieved [5, 15]. In this study, results on measures of prevention are similar to those obtained in a recent study led in Cameroon and in Gabon [11, 16].

Not surprisingly, this study showed a lower malaria prevalence in children aged 5 years or under and women aged 15–49 years (13.6%) than that found in previous studies in other rural areas (Makokou 53.6%, Lastoursville 79.5%, Oyem 44.2%) [6, 17]. This difference could be due to the fact that targeted population was: children ≤ 5 years old and women aged 15–49 years old in the general population who were mostly asymptomatic, whereas in other studies, the population tested was based in hospitals for some febrile patients and involved mostly children. The low prevalence of malaria infection observed could also be associated with the high level of knowledge of preventive measures in the study area. [18]. The distribution of malaria prevalence among the 5 departmental capitals of Nyanga Province was quite varied. The departmental capitals of Tchibanga and Moabi had a prevalence of 18.7 and 14.8%, respectively, the highest prevalence for malaria parasite. Departmental capitals of Moulengui-Binza and Mabanda both showed parasite prevalence lower than 10% while in departmental capitals of Mayumba, malaria parasite was not detected at all. This variation between departmental capitals could be explained by some factors, such as the population density. In fact, Tchibanga, which is the main town of Nyanga Province comprises more than half the total population in that geographical area. It was surprising that none of 188 participants that were tested in Mayumba was positive for malaria infection since Gabon is hyperendemic for malaria.

The data revealed no relationship between preventive measures (ITNs and insecticides) and malaria prevalence in the study sites, perhaps because overall net use was very high, as assumed in another study [19]. This finding may also indicate that *Plasmodium* infections likely occur during non-sleeping hours.

## Conclusion

High malaria prevalence is found in the departmental capital cities of Nyanga Province. This study reveals that respondents have a high knowledge of mode of transmission, malaria symptoms and preventive measures for the disease. Despite this high level of knowledge of the disease and its preventive measures, the incidence of malaria remains relatively high in this rural community highlighting the need for other types of interventions such as vector control and promoting access to malaria testing and treatment.

## Supplementary information

**Additional file 1.** Questionnaire.

## Data Availability

No applicable.

## Abbreviations

WHO: World Health Organization
ACT: Artemisinin-based combination therapy
RDT: Rapid diagnostic test
ITN: Insecticide-treated bed nets
LLN: Long-lasting insecticide-treated nets
NGO: Non-governmental organization
MCDI: Medical Care Development International
CIRMF: Centre International de Recherches Médicales de Franceville
