# Supplementary material for: Malaria in children and women of childbearing age: infection prevalence, knowledge and use of malaria prevention tools in the province of Nyanga, Gabon
Source: Malar J. 2020 Nov 2;19:387. doi: 10.1186/s12936-020-03411-5 (PMC7607695; doi:10.1186/s12936-020-03411-5)
Supplement: Supplementary file 1 — Additional file 1. Questionnaire. [file 12936_2020_3411_MOESM1_ESM.docx]

Centre International de Recherches Médicales de Franceville
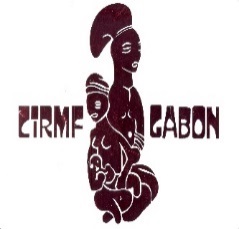


BP : 769 Franceville Gabon

Tel : (241) 67 70 92/67 70 96

Fax :(241) 67 72 95/67 79 30

**Questionnaire: patient sheet**

**Evolution of the characteristics of malaria infection in Nyanga, south of Gabon, March 18th to 27th 2018**

**INFORMED CONSENT FORM**

Name :…………………………………………Surname :…............................................

Name and surname of parent or legal guardian (for minors) :.………………………….

Tel :………………………………...

**OBJECTIVE OF THE STUDY**

Malaria is currently one of the main causes of mortality in the world, particularly in countries located in intertropical areas. Malaria is much more present in rural areas and in remote locations. Data on malaria in the Nyanga province are still unavailable. It is therefore necessary to initiate studies to understand the impact of malaria in this province, which is one of the least developed in Gabon. The objective of this study is to study the characteristics of malaria in the Nyanga province.

**PROCEDURE**

After your written consent, you will be examined and several questions will be asked. Then, a member of the team will take a blood sample and put it in a EDTA tube, which will allow us to do different types of exams. Blood sampling is a calculated risk, which can sometimes lead to complications in the form of a hematoma around the puncture area which will be eliminated after a few days. All the documents of the study will be given by the laboratory. All the collected data concerning you (or your child) will strictly be confidential.

**POTENCIAL BENEFITS OF THE** **STUDY**

Your participation to this study will help improve knowledge on the distribution of *Plasmodium* in the Nyanga province. The exams are free of charge. If you or your child have malaria, medication will be given to you. In this case, you will benefit from a specific monitoring until your complete recovery.

Investigator :……………………

Date :……………………

Signature of the person (or parent or legal guardian) Signature of the investigator

**ADDITIONAL INFORMATION ON THE PERSON**

1. Identification number :……………………………………………………

2. Date of sampling :…………………………………………………………………...

3. Place of collection :…………………………………………………………………….

4. Date of birth :………………………………………………………………………

5. Sex : Girl Boy

6. Place of residence :………………………………………….……Longitude° :……… ; Latitude° :…….; Altitude° :……..

7. Duration (Days, Weeks, Months, Years) :……………………….

8. Have you stayed outside your residence and traveled elsewhere in these last fourteen (14) days or last eight (8) weeks?

Yes Place of stay :…………………

No

9. Number of children < 5 years in the household :………………………………

10. Number of pregnant women in the household :………………………………

11. Number of months of pregnancy :……………………………………………………… 12. Use of IPT for pregnant women : IPT1 IPT 2 IPT3

13. Number of people in the household :…………………………………………

14. Profession (or profession of the father for minors) :………………………………

15. Profession (or profession of the mother for minors) :……………………………

**INFORMATION AND EDUCATION ON MALARIA**

16. Have you already heard of Malaria? Yes No

17. If yes, how is it transmitted? By ingesting dirty water By walking under the rain Through a mosquito bite By having unprotected sexual intercourse

18. What are the symptoms which can make you think of malaria?

Fever Cough

Fatigue Diarrhea Ache Headache Vomiting

Stomachache Others…………………………………………………………………………………….

19. When you feel you have malaria what do you do?

Do you consult a doctor? Yes No

20. Do you go to a drugstore then take medication? Yes No If yes, which medication do you take?................................................................................

21. Do you take herbal tea? Yes No

22. What means of fighting against malaria do you know?

- Use of bed nets?

- Vaccination

- Spray of insecticides ?

- Washing your hands before every meal

- Environmental sanitation?

- Others……………………………………………………………………………………

23. If someone suffers from malaria, what is the best medication to cure him according to you?.......................................................................................................

24. Have you heard or seen a message in relation to malaria in the last six (6) months? Yes No

25. Where have you heard or seen this message?

…………………………………………………………………………………………………

26. How many times must a pregnant woman take IPT to protect herself from malaria?.............................................................................................

27. In which months (or trimester) of the pregnancy must a pregnant woman begin to take IPT to protect herself from malaria?……………………

You do not know the answer: ……………………..

28. Did you sleep under a bed net last night? Yes No

29. Are insecticide treated bed nets (ITNs) free for pregnant women in public health structures? Yes No

30. Did you receive a bed net in one of your antenatal visits? Yes No

31. Do you use insecticides? Yes No

32. Which types of ventilation do you use?

Fans Air conditioning None

33. Is Intermittent Preventive Treatment (IPT) free of charge for pregnant women in public health structures? Yes No

**CLINICAL SIGNS AT THE TIME OF SAMPLING**

34. Fever Yes (T°……..) History of fever in the last 14 days No Yes

35. Complaint : Yes No If yes : Chills Nausea Vomiting Headache Polyarthralgia Asthenia

Others :…………………………………………………………………………….

36. Treatment before consultation : Yes No

**BIOLOGICAL CRITERIA**

37. Rapid diagnostic test for *Plasmodium* : Positive for plasmodium *falciparum* Positive for non-*falciparum* plasmodium Positive for mixed malaria Negative Invalid result

38. Blood collection : NFS Not carried out

**DIAGNOSTIC AND CONCLUSION**

39. Malaria : Simple Moderate Severe None

40. Prescribed treatment :...…………………………………………………………………

**comments**

……………………………………………………………………………………………………………………………………………………………………………………………….
